# Variation of rectal temperature in dogs undergoing 3T-MRI in general anesthesia

**DOI:** 10.3389/fvets.2023.1156773

**Published:** 2023-07-25

**Authors:** Frauke Paul, Michaele Alef

**Affiliations:** Department for Small Animals, Faculty of Veterinary Medicine, University of Leipzig, Leipzig, Germany

**Keywords:** hypothermia, body temperature, dog, hyperthermia, diagnostic imaging

## Abstract

**Objectives:**

Managing body temperature during MRI scanning under general anesthesia poses challenges for both human and veterinary patients, as many temperature monitoring devices and patient warming systems are unsuitable for the use inside an MRI scanner. MRI has the potential to cause tissue and body warming, but this effect may be counteracted by the hypothermia induced by general anesthesia and the low ambient temperature usually encountered in scanner rooms. This study aimed to observe temperature variations in dogs undergoing MRI under general anesthesia.

**Materials and methods:**

In this prospective observational study, client-owned dogs scheduled for 3-Tesla MRI under anesthesia between February and October 2020 at a veterinary teaching hospital were eligible for enrollment. Recorded data included breed, body mass, body condition score, age, fur quality, pre- and post-MRI rectal temperatures, time in the MRI room, scan area and coil used, application of contrast medium, choice of anesthetic agents, use of blankets, and infusion therapy. Group comparisons were conducted using the Mann–Whitney U-test or Kruskal-Wallis test, with *p* < 0.05 considered significant.

**Results:**

In total 171 dogs met the inclusion criteria. The median body temperature at admission was 38.4°C (IQR 38.1–38.7°C). The median body temperature before MRI was 38.2°C (IQR 37.8–38.6°C), and the median temperature after the MRI scan was 37.7°C (IQR 37.238.2°C) resulting in a median temperature difference (∆T) before and after MRI of - 0.6°C (IQR −0.8–−0.1°C). The median duration of MRI scans was 49 min (IQR 38–63 min). A temperature loss of more than 0.1°C was observed in 121 (70.8%) dogs, 29 (16.9%) dogs maintained their temperature within 0.1°C, and 21 (12.3%) dogs experienced a temperature increase of more than 0.1°C. Factors associated with a higher post-MRI temperature included greater body mass, medium or long fur, and the application of α_2_- receptor-agonists.

**Conclusion:**

Dogs undergoing MRI under general anesthesia are likely to experience temperature loss in the given circumstances. However, in larger dogs and those with much fur, an increase in body temperature is possible and more common than generally anticipated, although clinically insignificant in most cases.

## 1. Introduction

MRI, as a diagnostic imaging modality, is increasingly available in veterinary medicine, while access to high-field MRI with 3 Tesla is also expanding (1). A successful MRI requires the patient to remain still to ensure optimal image quality; therefore, animals need at least deep sedation or general anesthesia (2). Consequently, the situation in veterinary medicine is similar to that in human pediatrics, with uncertainties surrounding the impact of tissue warming effects due to MRI and temperature loss due to general anesthesia (3).

Thermoregulation is significantly impaired by general anesthesia (4). Hypothermia is the most common complication in small animal patients undergoing general anesthesia, with incidence rates as high as 83.6% (5, 6) and 63.8% in patients undergoing anesthesia for thoracolumbar hemilaminectomy (7). Performing an MRI alone or during the same anesthesia as surgery has been identified as a significant risk factor for hypothermia (6, 7). Hypothermia can lead to severe and life-threatening consequences, including an increased incidence of delayed recovery, wound infections, coagulation deficiency, bradyarrhythmia, and hypotension (8, 9).

However, hyperthermia after general anesthesia has also been reported. In a study, hyperthermia, defined as a rectal temperature exceeding 39.5°C, was observed in 2.9% of dogs undergoing various procedures under general anesthesia, but no further discussion on the reasons for its occurrence was provided. Nonetheless, this study associated diagnostic imaging of any kind with temperature loss (5). Another study examining anesthetic complications in dogs undergoing neurologic surgery reported that 20.5% of patients exhibited hyperthermia (defined as a body temperature > 39.0°C), with the risk of hyperthermia increasing with anesthesia duration and the use of α_2_- receptor-agonists (7). A study investigating temperature change in dogs under general anesthesia found that 9% of dogs experienced an increase in temperature during general anesthesia, which was associated with the type and lack of opioid as well as anesthesia duration (6). Depending on the degree, hyperthermia can lead to severe consequences. Severe hyperthermia is defined as a rectal temperature higher than 40.5°C and can cause neurologic disfunction, acid–base disturbances as well as multi-organ failure (10, 11). Milder degrees of hyperthermia may not have as dramatic consequences but can manifest in symptoms such as panting and tachycardia, both unwanted complications during anesthesia and recovery phase, while not necessarily harmful.

MRI generates radio frequencies that are converted into heat upon absorption by the body, potentially affecting tissue and body temperature, especially when modern high-field scanners with field strengths of 3 Tesla or higher are used (3, 12, 13). In human pediatric patients, reports of increased core body temperature during MRI scanning have been shown, but could not been reproduced by other studies (2, 12–18).

While some authors mention the risk of temperature rise during MRI in veterinary patients (3, 19), the incidence and clinical relevance remain unclear due to a lack of data for veterinary patients (3).

Continuous monitoring of body temperature within the MRI scanner is challenging and expensive since most temperature monitoring systems are unsuitable for use within the magnetic field (2). Therefore, measuring temperature before and after the MRI scan is an accepted surrogate method to assess temperature changes during the procedure (2, 7, 12, 13, 20, 21). Implementing MRI-compatible temperature monitoring devices would only be justified if significant temperature variations occur regularly. As concerns regarding hypo- and hyperthermia may influence a clinician’s decision to perform diagnostic procedures, understanding the influence of MRI scanning on body temperature is crucial.

Therefore, the objective of this study was to investigate the incidence, magnitude, and nature of body temperature variations as well as risk factors associated with those changes in dogs undergoing high-field MRI scanning under general anesthesia.

## 2. Materials and methods

This prospective observational study included dogs that underwent MRI under general anesthesia between February and October 2020 at the Small Animal Teaching Hospital, University of Leipzig. Ethical approval (EK 15/2021) was obtained from the local ethics committee, and owners provided consent for the use of anonymous data for research purposes upon hospital admission.

Dogs were excluded from the study if they had underlying diseases that could cause fever, if they were hypo- or hyperthermic during the pre-anesthetic examination at admission (with normal rectal temperature defined as between 37.4–39.2°C) (8), if there was suspicion of rectal disease, if the time span between anesthesia induction and the start of the MRI scan exceeded 15 min, or if any procedure potentially influencing body temperature (e.g., radiography or computed tomography) was performed prior to the MRI. In cases where an animal was scheduled for multiple MRIs during the study period, only the first MRI was included.

The choice of anesthesia and analgesia protocols was left to the discretion of the individual anesthetists managing the cases, including the selection of the breathing circuit, use of mechanical ventilation, and fluid therapy.

The MRI was performed with a 3-Tesla MRI scanner (Philips Ingenia 3T, Philips Healthcare, Hamburg, Germany). The MRI room maintained an environmental temperature of 20°C controlled through air conditioning, while the scanning room’s humidity was kept around 60%. Room temperature was recorded once daily during the study period using an analog device.

During the MRI scan, dogs were positioned on a plastic mat without active warming methods. In some cases, blankets were used to cover the patients. Anesthesia was maintained using Isoflurane (Isofluran CP, CP Pharma GmbH, Burgdorf, Germany) in oxygen or oxygen/medical air, administered through an anesthesia machine (Datex Ohmeda Aestiva/5 MRI, GE Healthcare Datex-Ohmeda GmbH, Freiburg, Germany) with a circle system (either pediatric in patients up to 15 kg body mass or adult rebreathing system in larger patients). Oxygen saturation, heart rate, capnography, and inhalational gas analyses were continuously monitored using a MRI-compatible multi-parameter monitor (Datex/Ohmeda S/5, GE Healthcare Datex-Ohmeda GmbH, Freiburg, Germany).

Rectal temperature measurements were taken upon arrival in the MRI preparation room using a commercially available veterinary digital thermometer with an accuracy of +/−0.1°C stated by the manufacturer (Kerbl, Albert Kerbl GmbH, Buchbach, Germany). The same thermometer was used for temperature measurements before and after the MRI scan in all patients throughout the study. The thermometer was gently inserted into the rectum as deeply as possible without resistance. Care was taken to ensure full contact between the thermometer and the rectal mucosa, minimizing interference from feces during the measurement. No cover or lubricant was used for temperature measurements under general anesthesia. The thermometer was cleaned and disinfected between each use. Measurement began after successful placement of the thermometer in the rectum and ended when the thermometer indicated completion with an acoustic signal.

After temperature measurement, the dogs were positioned on a plastic mat within the MRI scanner, and a stopwatch was started. Rectal temperature was measured again upon leaving the MRI scanner, and the stopwatch was stopped.

For each patient, the following data were recorded: breed, sex, body mass (kg), body condition score (BCS) on a scale of 1–5, subjective assessment of fur quality (short/medium/long), total MRI scan duration (minutes), use of *α*_2_- receptor-agonists (yes/no) or acepromazine (yes/no), use of ketamine (yes/no), lidocaine (yes/no), opioid administration (yes/no), and if applicable, the type of opioid used (L-methadone/butorphanol), use of propofol (yes/no), and use of alfaxalone (yes/no). Furthermore, diagnostic imaging data was reviewed for the scanned body region, the coil used, and the use of contrast medium (if applicable).

### 2.1. Statistics

Statistical analyses were conducted using R (version 4.1.3). To enhance readability, all data is presented as median and interquartile range (Md, IQR), regardless of whether normal distribution was confirmed.

Dichotomous data was compared using the Mann–Whitney U-Test, while the comparison of more than two nominal variables was conducted through the Kruskal-Wallis test, followed by Dunn pairwise comparison, with *p* < 0.05 considered significant.

Subsequently, a stepwise linear regression analysis was performed to identify factors associated with temperature change after examining group differences.

Due to the lack of reported data for veterinary patients, no *a priori* sample size estimation was conducted before implementing this study. Instead, the study duration was guided by similar studies conducted in human children, which proceeded accordingly.

## 3. Results

**Figure 1:**
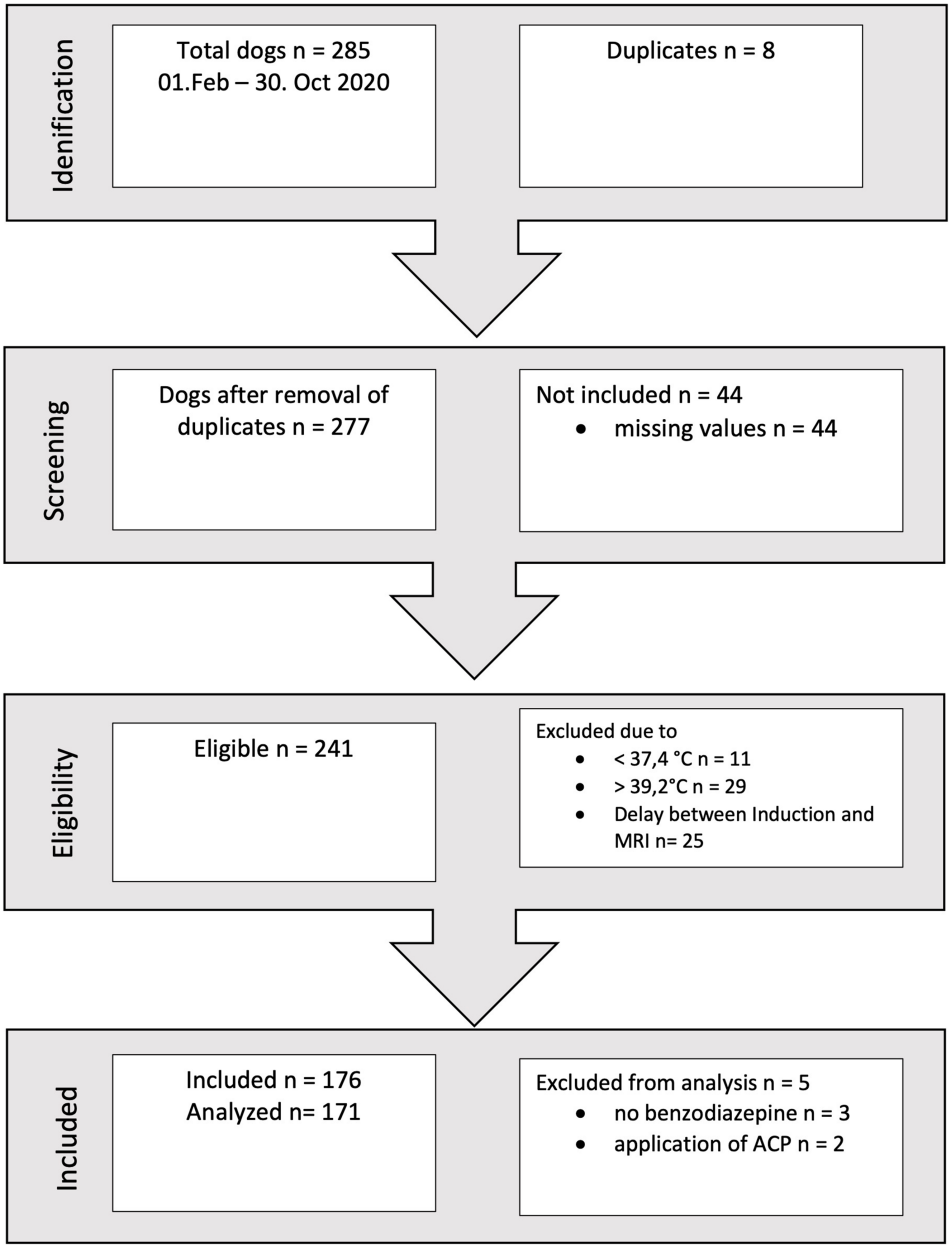
Flowchart of inclusion.

During a ten-month period, a total of 176 records met the inclusion criteria (refer to Figure 1). Following a thorough assessment of all parameters, two dogs were excluded as they had received acepromazine, and three dogs were excluded due to the absence of benzodiazepine administration. These exclusions were made because the respective patient subgroups were deemed too small to permit further analysis.

The remaining 171 dogs had a body mass between 2 and 83 kg (Md 15 kg, IQR 11–28.5 kg), the age ranged from 0.2–14.3 years (Md 6.5 years, IQR 3.5–8.5 years). Dogs of 46 different breeds were included as listed in Table 1. While French Bulldogs were clearly overrepresented (21.6% of all dogs), the broad variety of breeds in the study group precluded analysis of breed differences. The majority of dogs (60.2%, 103/171) was of ideal weight (BCS 3/5), underweight (BCS 2/5) was present in 19.3% (33/171) and overweight (BCS 4/5) in 16.4% (28/171). Only a small number of 4.1% (7/171) dogs were considered obese (BCS 5/5). There was no association between BCS and temperature.

**Table 1:** Summary of breeds included into the study.

Breed	Number
French Bulldog	37
Mix Breed	34
German Shepherd	9
Labrador Retriever	8
Pug Dog	7
Beagle	6
Dachshound	6
Golden Retriever	5
Boxer	4
Miniature Schnauzer	4
Australian Shepherd Dog	3
Chihuahua	3
Cocker Spaniel	3
Yorkshire Terrier	3
Bearded Collie	2
Bolonka	2
Dalmatian	2
English Bulldog	2
Great Dane	2
Jack Russel Terrier	2
Maltese Dog	2
Akita Inu	1
Australian Cattle Dog	1
Bichon Frisee	1
Border Collie	1
Cane Corso	1
Coton de tular	1
Entlebucher Mountain Dog	1
Great Swiss Mountain Dog	1
Hanover Hound	1
Landseer Dog	1
Magyar Vizla	1
Malinois	1
Miniature Bullterrier	1
Norwegian Buhund	1
Old English Bulldog	1
Pekinese	1
Podenco	1
Poodle	1
Portuguese Water Dog	1
Rhodesian Ridgeback	1
Russian Toy Terrier	1
Saluki	1
Shorty Bulldog	1
St. Bernard	1
West Highland White Terrier	1
Total:	171

107 dogs were male (77 intact, 30 neutered) and 64 dogs were female (41 intact, 23 neutered). There was no correlation between temperature and sex, therefore male and female animals were analyzed together.

Median body temperature at admission was 38.4°C (IQR 38.1–38.7°C). Median body temperature before MRI was 38.2°C (IQR 37.8–38.6°C) and median temperature after MRI scan was 37.7°C (IQR 37.2–38.2°C). Median difference in temperature before and after MRI (∆T) was −0.6°C (IQR -0.8–−0.1°C). MRI duration ranged from 14 min to 109 min (Md 49 min, IQR 38–63 min). Temperatures are also summarized in Table 2. 124 (73.5%) dogs had short fur, 36 (21.1%) had medium fur and 11 (6.4%) had long fur.

**Table 2:** Summary of temperatures before and after MRI and temperature changes for all dogs, sorted by fur quality and for dogs undergoing head MRI sorted by coil used.

		Temperature before MRI	Temperature after MRI	∆T
All dogs		38.2 (37.8–38.6)	37.7 (37.2–38.2)	−0.6°C (−0.8–−0.1°C)
Fur quality	Short	38.1 (37.7–38.5)	37,5 (37.1–38.0)	−0.6°C (−0.9–−0.2°C)
	Medium	38.4 (38.2–38.8)	38.0 (37.7–38.4)	−0.4°C (−0.7–0°C)
	Long	38.4 (38.1–28.6)	38.3 (38.3–38.8)	+0.1°C (−0.3–0.3°C)
Coil used for head MRI	Small extremity	38.0 (37.9–38.4)	37.4 (37.2–37.6)	−0,7 (−1–−0.4)
	Knee	38.3 (38.0–38.6)	37.7 (37.5–38.0)	−0.7 (−0.9–−0.2)
	Head	38.2 (37.9–38.5)	38 (37.5–38-3)	−0.4 (−0.8–0)

All values are displayed as median (interquartile range) in °C.

Dogs with short fur had a median body temperature of 38.1°C (IQR 37.7–38.5°C) before MRI and a median body temperature of 37.5°C (IQR 37.1–38.0°C) after MRI and a median ∆T of – 0.6°C (−0.9–−0.2°C). Dogs with medium fur had a median temperature before MRI of 38.4°C (IQR 38.2–38.8°C) and a median temperature after MRI of 38.0°C (IQR 37.7–38.4°C) and a ∆T of – 0.4°C (IQR −0.7 −0°C) and dogs with long fur had a median temperature before MRI of 38.4°C (IQR 38.1–28.6°C) and a median temperature after MRI of 38.3°C (IQR 38.3–38.8°C) and a ∆T of +0.1°C (IQR −0.3 – 0.3°C). The differences in temperature after MRI between dogs with short and medium fur (*p* < 0.001) and short and long fur are statistically significant (*p* = 0,004), as well as the ∆T between dogs with short and medium fur (*p* = 0.04) and short and long fur (*p* = 0.004). Temperatures are also summarized in Table 2.

121 (70.8%) of the dogs lost more than 0.1°C (with −2.3°C being most pronounced temperature decrease), 29 (16.9%) dogs kept their temperature +/− 0.1°C constant and 21 (12.3%) dogs gained more than 0.1°C (with +0.9°C being the most pronounced temperature increase).

91 (53.2%) dogs underwent an MRI of the body (88 spine, one knee, one shoulder), 61 (35.7%) dogs underwent an MRI of the head, and 19 (11.1%) dogs had both regions scanned. Median ∆T after head MRI was −0.7°C (IQR −0.9 – −0.4°C), after body MRI −0,4°C (IQR −0.8 − 0°C) and after scanning of two regions −0.7°C (IQR −1−−0.1°C). While there was no significant difference in ∆T between head and body scanning (*p* = 0.12), dogs that underwent a head MRI had a lower temperature after the MRI of 37.5°C (IQR 37.2–38°C) compared with dogs that underwent body scanning (Md 37.8°C, IQR 37.3–38.3°C) (*p* = 0.05).

Of the dogs that underwent MRI of the body all except 2 dogs were examined in with an anterior/posterior body coil, in one patient a small extremity coil was used for a scan of the knee and in one dog with a body mass of 2 kg a small extremity coil for a MRI of the spine.

Of the dogs that had an MRI of the head a human small extremity coil was used in 31 dogs, a coil designed for human knees was used in 27 dogs and a human head coil was used in 3 cases. As the selection of the coil is based on the size of the patient, the body mass of the dogs examined in the three different coils differed significantly (*p* < 0.001), but there was neither a significant difference in the temperatures measured at the different time points nor regarding ∆T (Table 2).

104 dogs received contrast media (Dotarem, Gadotersäure, 0.5 mmoL/mL, 0.2 mL/lg, Guerbert GmbH, Sulzbach, Germany). The median ∆T in the dogs that received a contrast medium was −0.6°C (−0.9 - −0.1°C) while the median ∆T in the non-receiving group ∆T was −0.5°C (−0.8–−0.1°C). This difference was not statistically significant (*p* = 0.33). The median temperature after MRI scanning was significantly higher (*p* = 0.04) in the non-receivers (Md 37.9°C, 37.5–38.5°C) than in the receivers (Md 37.5°C, 37.2–38.2°C). The median MRI duration in the dogs receiving contrast medium was shorter (Md 34 min, 26–44 min), than in the non-receiving group was (Md 57 min, 49–68 min) (*p* < 0.001).

There was a negative correlation between median ∆T and MRI duration ( $r_{sp}=$ −0.138, *p* = 0.07) and a positive correlation between median ∆T and body mass (Spearman correlation 0.235; *p* = 0.002) and body mass and temperature after MRI (Spearmann correlation 0.334; *p* < 0.001). There was no statistically significant correlation between temperature and age.

In group wise comparisons, the use of *α*_2_- receptor-agonists and ketamine was associated with a higher temperature after MRI; while the use of butorphanol compared to no opioid use led to a lower temperature after MRI as well as the use of propofol or alfaxalone. The influences of different anesthetic agents and infusion applied to the patients are shown in Table 3.

**Table 3:** Group-wise comparison of different anesthetic agents applied to the patient population.

	Yes	No	*p*-value
α_2_- receptor agonist	*n* = 92 (53.8%)	*n* = 79 (46.2%)	
∆T	−0.4 (−0.8–0.1)	−0.6 (−0.9–−0.3)	**0.007**
T pre MRI	38.4 (38.0–38.4)	38.0 (37.7–38.4)	**<0.001**
T post MRI	38.0 (37.6–38.4)	37.5 (37.0–37.8)	**<0.001**
Ketamine	*n* = 108 (63.2%)	*n* = 63 (36.8%)	
∆T	−0.5 (−0.8–0)	−0.7 (−0.9–−0.4)	0.07
T pre MRI	38.3 (37.8–38.7)	38.1 (37.8–38.5)	0.07
T post MRI	37.8 (37.3–38.3)	37.5 (37.1–37.9)	**0.02**
Opioid	*n* = 103 (60.2%)	*n* = 68 (39.8%)	
∆T	−0.5 (−0.8–0)	−0.6 (−0.8–−0.1)	0.21
T pre MRI	38.1 (37.7–38.4)	38.4 (38.0 38.7)	**0.04**
T post MRI	37.6 (37.2–38.2)	37.7 (37.4–38.3)	0.29
L-Methadone	*n* = 61 (35.7%)		
∆T	−0.6 (−0.8–−0.1)			0.50
T pre MRI	38.2 (37.7–38.7)			0.31
T post MRI	37.8 (37.2–38.2)			0.20
Butorphanol	*n* = 42 (24.6%)			
∆T	−0.7 (−0.9–−0.2)			0.08
T pre MRI	38.1 (37.8–38.5)			0.22
T post MRI	37.5 (36.9–37.9)			**0.007**
Alfaxalone	*n* = 15 (8.8%)	*n* = 156 (91.2%)	
∆T	−0.6 (−1.2–−0.5)	−0.6 (−0.8–−0.1)	0.08
T pre MRI	38.0 (37.6–38.7)	38.2 (37.9–38.6)	0.44
T post MRI	37.4 (37.0–37.7)	37.7 (37.3–38.2)	**0.04**
Propofol	*n* = 98 (57.3%)	*n* = 73 (42.7%)	
∆T	−0.6 (−0.9–0.2)	−0.5 (−0.8–0)	0.07
T pre MRI	38.2 (37.8–38.5)	38.2 (37.8–38.8)	0.07
T post MRI	37.5 (37.1–38.0)	37.8 (37.4–38.4)	**0.01**
Lidocaine	*n* = 6 (3.5%)	*n* = 164 (95.9%)	
∆T	−0.6 (−0.8–0)	−0.6 (−0.8–−0.1)	0.78
T pre MRI	38.1 (37.8–38.3)	38.2 (37.8–38.6)	0.44
T post MRI	37.8 (37.5–37.9)	37.7 (37.2–38.3)	0.99
Infusion	*n* = 9 (5.3%)	*n* = 162 (94.7%)	
∆T	−0.7 (−0.8–−0.3)	−0.6 (−0.8–0.1)	0.64
T pre MRI	38.7 (38.2–38.8)	38.2 (37.8–38.6)	0.1
T post MRI	37.7 (37.7–38.2)	37.7 (37.2–38.2)	0.64

Variables were assessed dichotomic and number of animals in either group are given as number (*n*) and percentage. Data are presented in °C as median (interquartile range), *p* < 0.05 is considered significant. Bold values represent *p*-values <0.05.

The use of blankets for covering the patients was documented in 6 cases, which all had a negative ∆T.

When using the definition of normal temperature of 37.4–39.2°C for awake dogs as a threshold in anesthetized dogs as well, 28% (48/171) dogs were hypothermic, 68% (116/171) were within the limits of normothermia and 4% (7/171) were hyperthermic. These 7 animals are summarized in Table 4.

**Table 4:** Patient characteristics of the seven patients with a post-MRI rectal temperature of more than 39.2°C.

Breed	Mass (kg)	Age (years)	Fur	BCS (1–5)	admission Temp	Temp preMRI	Temp post MRI	∆T °C	MRT duration	Region 1	Region 2
Norweg. Buhund	14.7	5.81	medium	2	39	39	39.7	0.7	45	Spine	
Malinois	30	10.23	short	3	39.1	39.1	39.6	0.5	81	Spine	elbow
German Shepherd	42	11.75	medium	3	38.6	39.3	39.5	0.2	69	Spine	
Landseer	83	3.29	medium	4	38.8	38.8	39.4	0,6	106	Spine	Head
Bernese Mountain Dog	63	4.76	medium	4	38.7	38.8	39.4	0.6	66	Spine	
Bearded Collie	21	10.88	long	3	38.6	38.4	39.3	0.9	57	shoulder	
Australian Cattle Dog	17	5.65	short	3	38.5	39.4	39.3	−0.1	32	spine	

The initial regression model consisted of the following explanatory variables: body mass, fur quality, temperature at pre-anesthetic exam, pre-MRI temperature, total scan time, application of contrast-medium, α_2_- receptor-agonist, opioid and ketamine. Of these, temperature at pre-anesthetic exam, application of contrast medium, opioid and ketamine were excluded from the model in a stepwise process, as they were not associated with post-MRI temperature at the level of significance.

The final linear regression contains five variables (temperature before MRI, body mass, medium or long fur, duration of MRI, administration of *α*_2_- receptor-agonist) significantly accounting for 61% of the temperature variation after MRI scanning. The administration of *α*_2_- receptor-agonists was kept in the regression model according to former described effects and results of groups-wise comparisons despite lack of significance in the regression (Table 5).

**Table 5:** Linear regression analysis of associations between body temperature after MRI and patient or procedural factors.

	Regression coefficient
Temperature before MRI	0.87 **
Body mass	0.01 **
Medium fur	0.24 *
Long fur	0.46**
Duration of MRI	−0.01 **
Administration of alpha-2-agonist	0.15
R2	0.62
Adjusted R2	0.61

*p < 0.05 **, *p* < 0,01.

## 4. Discussion

In our study sample, the incidence of temperature loss was 70.8% overall. However, we found that 16.9% of dogs managed to maintain their body temperature, while 12.3% experienced a gain of body temperature. The occurrence of body temperature reduction during general anesthesia has been widely recognized as a well-known risk, as described in previous studies (6, 7, 20, 21). Several studies have identified diagnostic imaging as a contributing factor to this phenomenon (5–7).

In our study group, the extent of heat loss was lower than previously reported, and a higher number of dogs managed to maintain or even increase their body temperature. This finding contrasts with a recent study that examined factors influencing changes in rectal temperature in dogs under general anesthesia for various procedures. The aforementioned study reported a positive ∆T in 9% of dogs and a ∆T of 0 in 2% (6). It is worth noting this difference considering that active warming techniques were used in many of the patients in that study, whereas no active warming was used in our study sample.

Among the 49 dogs undergoing MRI at five different institutions describe by Clark-Price et al. (6), the median ∆T was −1.5°C, ranging from −0.1 to −5.3°C. Comparing the characteristics of the dogs in that study to our study population, which displayed a similar degree of variation in most variables, it is noteworthy that none of the dogs in our sample exceeded a ∆T of −2.3°C. Unfortunately, the authors did not provide information regarding the specific types of MRI scanners used or the environmental conditions in their study.

Another study (7) also identified the performance of MRI as a significant risk factor for hypothermia, together with hypotension. This study focused on peri-anesthetic complications during anesthesia for spinal surgery and described a relative high incidence of hyperthermia in this patient population but associated with duration of general anesthesia and premedication with *α*_2_-receptor agonists.

We observed that dogs with higher body mass and those with medium or long fur had a reduced risk of heat loss. The association between increased body mass and a decreased likelihood of hypothermia has been previously documented and can be attributed to a lower body surface area, resulting in reduced heat loss through radiation (5, 7). In humans, patients with a higher body mass index have been shown less affected by peri-anesthetic hypothermia (22) and this is also proposed to be true for dogs (11). While BCS was assessed in our study no influence could be detected, but majority of dogs were of normal weight.

In our study sample animals with medium to long fur experienced less heat loss, a finding that might be considered analogous to the use of insulation materials, as proposed by Özer et al. (21). This observation may seem intuitive, yet it can become a critical factor when performing MRI scans on large dogs with medium or long fur.

The evaluation of fur quality in our study was subjective, leading to a possible uniformity in identifying and categorizing dogs with short fur. However, potential inconsistencies may have occurred in assigning animals to the medium or long fur categories. This inconsistency is because the insulating potential of fur might not solely dependent on length, but also on additional factors such as thickness and the presence of an undercoat.

Thus, for animals with highly insulative fur, implementing cooling procedures may prove beneficial. Such methods may include adjusting the MRI fans, increasing fresh gas flows, modifying fluid therapy, and tailoring the anesthetic protocol to suit individual animals’ needs.

It is important to note that most anesthetic agents have some impact on thermoregulation (11). The use of α_2_-receptor agonists has yield varying effects on body temperature during general anesthesia. Some authors have demonstrated a temperature-preserving effect (19), while others have identified it as a risk factor for temperature loss (6). In our study sample, dogs that received α_2_-receptor agonists tended to have higher body temperatures than those that did not. In dogs, α_2_-receptor agonists initially induce peripheral vasoconstriction which may reduce cutaneous heat losses (23). In our study, MRI was performed immediately after premedication and induction of anesthesia, making it plausible that this mechanism of heat preservation was present in most animals. However, this effect did not remain statistically significant in the regression model.

The effect of opioids on body temperature is influenced by the species. In dogs, the administration of opioids is typically associated with hypothermia or temperature loss. This effect is caused by reduced peripheral vasoconstriction and a reduction in the central thermoregulatory set point. Interestingly, the use of κ-opioid receptor agonists has been shown to prevent temperature loss compared to the use of full μ-opioid receptor agonists (6). This finding contradicts our own observation that dogs receiving butorphanol had slightly lower temperatures than those not receiving an opioid. However, this temperature difference was minimal and did not reach statistical significance in the regression model.

Regarding the choice of induction agent, we observed a minor impact on rectal temperature following MRI. Dogs induced with either propofol or alfaxalone had slightly lower body temperatures compared to those not induced with either drug, while induction with ketamine resulted in a slightly higher temperature after MRI. However, none of these effects remained statistically significant in the regression model. The effect of propofol on temperature has been described in both humans and dogs, attributed to enhanced heat loss through peripheral vasoconstriction (24). However, these findings could not be consistently reproduced in studies involving more diverse patient populations (6).

The body temperature in animals receiving contrast media (gadolinium) during an MRI was higher than in those who did not, but the scanning duration in the non-receiving group was also significantly shorter. Therefore, the scanning duration might confound this finding. The use of contrast media was included in the documentation as fever was mentioned as a possible side effect in the manufacturer’s information. In humans, the incidence of adverse effects after the administration of gadolinium is generally rare and most often occurs after repeated administration. Most common adverse reaction in humans are nausea and vomiting as well as allergy-like reactions (25, 26). Information about adverse reactions in small animals is scarce and mainly focuses on hemodynamic changes (27). No clinical signs of adverse reactions were noted in any animal in our study. Therefore, it seems unlikely that the administration of contrast media influenced body temperature, but rather, the temperature differences should be attributed to the varying scanning durations.

Severe hyperthermia is generally known to cause more rapid changes in vital parameters compared to hypothermia (11, 28, 29). The initial effects of increasing body temperature include hypercapnia, panting or resistance against the ventilator, elevated heart rate, and alterations in blood pressure. Therefore, close monitoring of heart rate, blood pressure, and capnometry can provide valuable clinical clues in patients experiencing significant heat stress, especially in cases where continuous temperature monitoring is not available.

In our study, the use of blankets was only observed in a small subgroup of dogs, all of which experienced temperature loss despite being covered. The impact of using plain blankets, with or without pre-warming, has been demonstrated to have minimal influence in both human and animal studies (30, 31). Hence, the influence of blanket usage in our study group was likely negligible.

In total, 7 dogs (4.1%) had a temperature of 39.2°C or higher, while 119 dogs (69.5%) had temperatures ranging from 37.4°C to 39.1°C. Additionally, 45 dogs (27.6%) had temperatures below 37.4°C at the conclusion of the MRI examination. There is no consensus regarding temperature thresholds under general anesthesia that are causally associated with a higher incidence of complications in small animals (6). However, some authors consider body temperatures between 37.0°C and 39.0°C as normal and use this range as a guide for implementing temperature management during clinical decision-making (7, 8, 32). Therefore, comparing results between studies becomes challenging, but it also reflects the clinical practice at our institution. Based on our findings, the changes in body temperature within our study sample were rarely clinically significant, and none of the patients experienced other relevant changes in vital parameters nor reached a potentially harmful temperature of 40.5°C (11). As a result, increasing body core temperature to a harmful level does not appear to be a major concern in dogs undergoing high-field MRI.

Other studies have reported lower temperatures after MRI, despite efforts to prevent heat loss using a heat-and-moist exchanger (20). One possible reason for this discrepancy could be the use of a non-rebreathing system, which may result in higher fresh gas flows. Additionally, the explicit selection of dogs with a body mass below 10 kg in this study may contribute to different outcomes. However, another study (21) that examined a population of larger dogs found sporadic cases where dogs either maintained their body temperature or exhibited a slightly higher temperature (+0.3°C) after the MRI. The use of heat insulation led to a higher mean body temperature after the MRI but did not alter the number of dogs with a positive temperature difference.

In our clinical study, fresh gas flows were not standardized and were not documented. Since high gas flows contribute to temperature loss, this factor may play a significant role in temperature differences (33). It is important to note that we used rebreathing circuits for all animals, which likely resulted in lower fresh gas flows compared to non-rebreathing circuits (20). However, making further comparisons with other studies is challenging as other authors also did not provide information on gas flow parameters (20, 21).

The presented study has several limitations that should be acknowledged. Firstly, due to its clinical and observational nature, the study encompassed a diverse range of patients, indications, and scan and anesthesia protocols. While this accurately reflects the real-world scenario encountered in veterinary practice, it hindered the analysis of specific coils used for different body parts. The use of the same coil for most spine scans and the substantial variability in patient size across the three different coils used for head MRI prevented a valid comparison between them.

Furthermore, it was not possible to categorize the MRI protocols based on their absorption rates. Reliable data from our MRI scanner were not available, and standardization of scanning protocols was challenging due to the wide variety of protocols used and the inclusion of patients who underwent multiple scans. This limitation has also been observed in several studies involving pediatric patients, despite their more standardized approach and stricter limitations on applied energy (2, 13, 16).

It would have been beneficial to assess the warming effects of MRI in relation to the energy applied to the body, considering the correlation with body mass and duration (2, 34, 35). In human patients, the specific absorption rate (SAR) is calculated by the scanner to quantify this effect. However, the scanner used in our study could not reliably calculate SARs for veterinary patients. Additionally, different coils used during MRI can have varying influences on local tissue warming. The impact of these local effects on whole-body temperature remains unclear and can exhibit significant variability. Various factors, such as local perfusion, tissue thermal conductivity, and microvascular blood flow, contribute to thermal equilibration within the body (34–36). For example, it is plausible that using the body coil during spine scanning may cause local tissue warming over a considerable portion of the animal’s body surface, potentially contributing to higher post-MRI temperatures compared to head MRI patients. This phenomenon might be more pronounced in animals than in humans since the use of an anterior/posterior body coil covers large portions of the animal’s body, which is a relatively rare situation in humans (36).

However, temperature variations observed may also be attributed to the different scanning durations in this patient group. In our study, spine MRI examinations were generally much shorter than head scans, and this information is not captured in our data. It is important to highlight that all animals who were hyperthermic at the end of the MRI had a body coil used during the examination, and these examinations were comparatively long (refer to Table 4).

Ideally, continuous monitoring of body temperature during the MRI scan would have been preferable, considering the known non-linear decline of body temperature during anesthesia (5). However, such continuous monitoring is not available at our hospital. Therefore, the standard practice in our hospital, as well as in many other medical institutions, is to measure rectal temperature just before entering and after leaving the MRI room.

Rectal temperature measurement has been shown to be a reliable estimate of body core temperature (37), with a repeatability of 0.12°C (38). It is important to consider that factors such as a relaxed anal sphincter, intestinal air, fecal masses, decreased muscle tone, and physical activity can all influence rectal temperature measurements. In our study, all dogs were under general anesthesia, which resulted in at least a moderate degree of muscle relaxation, potentially impacting the temperature measurements.

While rectal temperature measurement is generally accepted as a method with low training requirements, variations in measurements taken by different operators may still exist. In comparable studies involving children, tympanic or temporal artery thermometers are commonly used, and serial measurements and averaging of values are performed to account for measurement variance (2). We decided not to adopt a similar approach because non-invasive temperature devices other than rectal measurements have been shown to be less reliable in small animals (39) and repeated rectal temperature measurements could potentially cause irritation to the rectal mucous membrane and introduce measurement disturbances through repeated opening of the anus. However, we acknowledge that measurement variations due to the chosen method may be present, representing clinical rather than research standards. Similar pragmatic approaches to assess temperature variations have been employed in several previous studies (6, 20, 31).

This cohort study focused specifically on patients undergoing MRI without including a control group to assess the influence of fur quality in patients undergoing procedures other than MRI. This decision was made because we were unable to identify another clinically comparable patient group undergoing procedures under general anesthesia with a similar procedure length and room temperature but without active body warming. It would have been ethically unacceptable to intentionally induce hypothermia in any potentially comparable patient group by withholding warming measures.

In this study, dogs with hyperthermia and fever were excluded to eliminate influences that did not originate from the MRI scanning itself but rather from the patient’s underlying condition. However, it should be noted that hyperthermic patients might be at an increased risk of developing even higher body temperatures during MRI scanning and should be closely monitored for this. Unlike in human pediatric patients, hyperthermia is not always considered a contraindication for anesthesia in veterinary patients (7).

In conclusion, hypothermia was the most common finding after MRI scanning under general anesthesia. Smaller dogs were at a higher risk compared to larger dogs, and dogs with medium or long fur showed a protective effect against heat loss. It is important to note that the temperature loss during high-field MRI was smaller than anticipated, and in some cases, the body temperature could even be maintained or increased. Patients at risk should be closely monitored to ensure their well-being during the procedure.

## Data availability statement

The raw data supporting the conclusions of this article will be made available by the authors, without undue reservation.

## Ethics statement

The animal study was reviewed and approved by ethics committee of the Faculty of Veterinary Medicine, University of Leipzig. Written informed consent for participation was not obtained from the owners because no explicit informed consent was deemed necessary as this study was observational and no additional procedures or treatments were performed on the animals. Consent to use anonymous data for research purposes was obtained at admission to the hospital.

## Author contributions

All authors listed have made a substantial, direct, and intellectual contribution to the work and approved it for publication.

## Funding

The publishing of this article was funded by the Open Access Publishing Fund of Leipzig University supported by the German Research Foundation within the program Open Access Publication Funding.

## Conflict of interest

The authors declare that the research was conducted in the absence of any commercial or financial relationships that could be construed as a potential conflict of interest.

## Publisher’s note

All claims expressed in this article are solely those of the authors and do not necessarily represent those of their affiliated organizations, or those of the publisher, the editors and the reviewers. Any product that may be evaluated in this article, or claim that may be made by its manufacturer, is not guaranteed or endorsed by the publisher.
